# Comparative evaluation of time series models for predicting influenza outbreaks: application of influenza-like illness data from sentinel sites of healthcare centers in Iran

**DOI:** 10.1186/s13104-019-4393-y

**Published:** 2019-06-24

**Authors:** Leili Tapak, Omid Hamidi, Mohsen Fathian, Manoochehr Karami

**Affiliations:** 1 Department of Biostatistics, School of Public Health, Modeling of Noncommunicable Diseases Research Center, Hamadan University of Medical Sciences, Hamadan, Iran; 2 Department of Science, Hamedan University of Technology, Hamedan, 65155 Iran; 3 Office of Information Technology, Hamedan Electrical Power Distribution Company, Hamedan, Iran; 4 Department of Epidemiology, School of Public Health, Research Center for Health Sciences, Hamadan University of Medical Sciences, Hamadan, Iran

**Keywords:** Influenza, Outbreak, Public health surveillance, Support vector machine, Neural network, Random Forest

## Abstract

**Objective:**

Forecasting the time of future outbreaks would minimize the impact of diseases by taking preventive steps including public health messaging and raising awareness of clinicians for timely treatment and diagnosis. The present study investigated the accuracy of support vector machine, artificial neural-network, and random-forest time series models in influenza like illness (ILI) modeling and outbreaks detection. The models were applied to a data set of weekly ILI frequencies in Iran. The root mean square errors (RMSE), mean absolute errors (MAE), and intra-class correlation coefficient (ICC) statistics were employed as evaluation criteria.

**Results:**

It was indicated that the random-forest time series model outperformed other three methods in modeling weekly ILI frequencies (RMSE = 22.78, MAE = 14.99 and ICC = 0.88 for the test set). In addition neural-network was better in outbreaks detection with total accuracy of 0.889 for the test set. The results showed that the used time series models had promising performances suggesting they could be effectively applied for predicting weekly ILI frequencies and outbreaks.

**Electronic supplementary material:**

The online version of this article (10.1186/s13104-019-4393-y) contains supplementary material, which is available to authorized users.

## Introduction

Influenza like illness (ILI) or acute respiratory infections is considered of the most important causes of mortality worldwide. As a nonspecific respiratory illness, ILI is defined by having fever over 38 °C along with cough and/or pharyngitis [1] and is mostly caused by viral pathogens though bacterial etiology might sometimes be encountered as well [2, 3] triggering epidemic peaks during the winter by influenza virus and respiratory syncytial virus [2]. According to the World Health Organization (WHO), each year there are 5–10% and 20–30% new cases of adults and children respectively that are infected with influenza [3]. This leads to 3–5 million severe illnesses causing 250,000–500,000 deaths all over the world [4]. Influenza viruses cause epidemics and pandemics and can accelerate them. This can lead to hospitalization of a large number of susceptible people that in turn imposes economic difficulties on families and society via absence from work/school [4]. In developing countries including Iran, the consequences of epidemics and pandemics of ILI can be more sever due to resource shortages and poverty in health and nutrition expenditures.

Various statistical outbreak detection methods have been developed to detect aberrations of ILI like classical time series methods and machine learning techniques. “ILI as a proxy of influenza activity and influenza related outbreaks occurrence has been used by surveillance systems of influenza worldwide” [5]. A web based tool, FluNet, has been developed by WHO to monitor influenza (http://www.who.int/influenza/gisrslaboratory/flunet/en). Few studies have been conducted in Iran regarding ILI outbreak detection and forecasting future outbreaks as a time series data set using classical methods including exponentially weighted moving average [5] and cumulative sum [6]. Machine learning methods including support vector machine (SVM), artificial neural network (ANN) and random forest (RF) are among the most promising methods and algorithms that can be used by the influenza surveillance systems to detect outbreaks/changes in ILI activity. Several studies have shown that these techniques have promising performance in predicting future events and have greater prediction accuracy compared with the ARIMA in different fields of research including public health [7–11].

Forecasting future outbreaks of ILI is one of most challenging public health priorities and forecasting seasonal outbreaks has a very important role in the planning and management of ILI by early response to health events. Moreover, accurate detection of ILI outbreaks is essential for public health authorities to implement interventions effectively in controlling the outbreaks and would help to minimize the effect of diseases via taking preventive steps especially in developing countries like Iran [12]. Therefore, evaluating performance of different methods as the main tools for outbreak detection in public health surveillance systems using real data testing is necessary to provide a reliable detecting system in timely detection of ILI outbreaks. To the best of our knowledge, no study has been conducted on evaluating the performance of the SVM, RF and ANN (three most widely used machine learning technique) in forecasting ILI cases and outbreaks in Iran. So, this study aimed to investigate the prediction accuracy of the SVM, ANN and RF time series models in forecasting ILI frequencies and outbreaks in weeks-ahead using ILI data in Iran from January 2010 to February 2018. The results of this study may be useful for designing early warning system outbreaks.

## Main text

### Materials and methods

#### Data

**Fig. 1:**
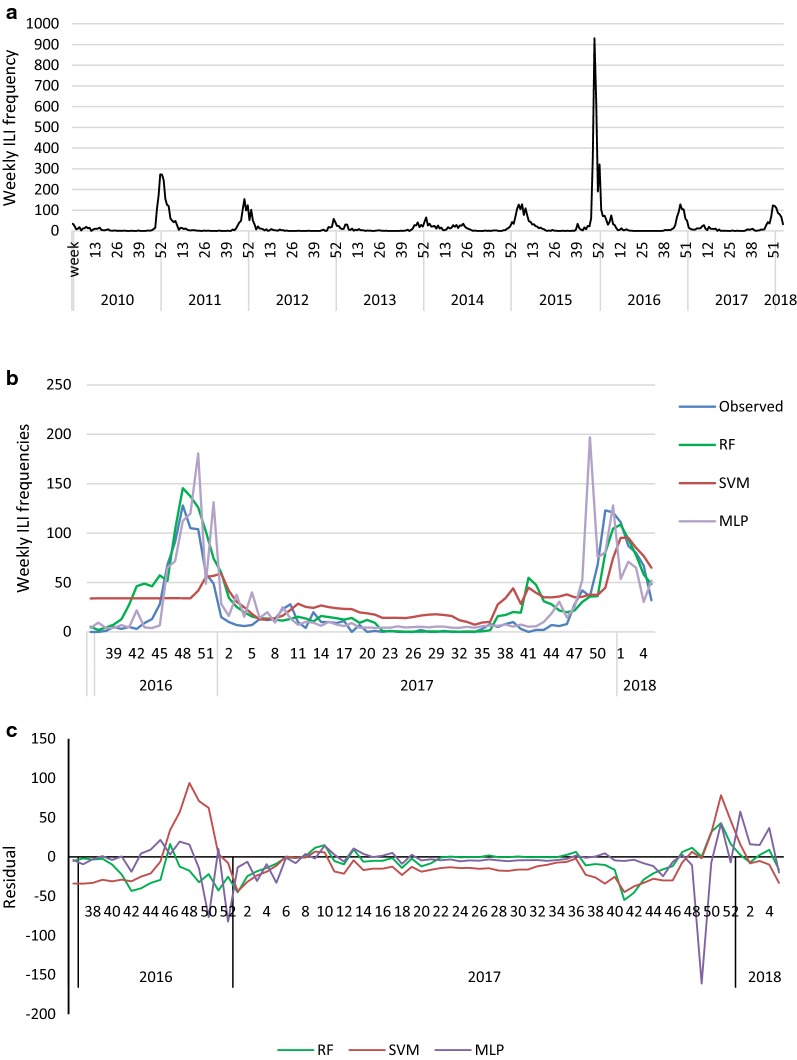
**a** Time series plot for observed ILI frequency over the study period of time; Y axis represents the weekly ILI rate; X axis represents time; **b** ILI prediction values and residuals (**c**) obtained using random forest time series (RFST), support vector machine (SVM) and artificial neural network (ANN) models along with the observed values over the testing set

We used the data related to all registered cases of ILI in Iran obtained from FluNet web base tool, World Health Organization from January 2010 to February 2018 (http://www.who.int/influenza/gisrs_laboratory/flunet/en). Information about the status of ILI activity including outbreak activity was also obtained from FluNet which is considered as the gold standard of influenza outbreak occurrence. Aggregated data related to 73483 ILI cases with fever more than 38 °C and cough that was started within 7 days were enrolled in this study. Figure 1a demonstrates the data, in which the Y axis represents the weekly ILI frequencies in Iran and the X is time axis represents outbreak time.

### Data analysis

In this study, the weekly ILI cases were considered as the response (output) variable and history observations and time of occurrence (year, season, week) were chosen as the predicator space. Considering *Y* as the current predicated point; the history observations was the sequence $X_{1} , \ldots ,X_{52}$, indicating the values of the preceding 52 observations before *Y*.

The SVM [13], ANN [14] and RF [15] time series models were applied to weekly reported counts of suspected cases of ILI to detect occurred outbreaks in Iran. As these methods are susceptible to overfitting problem, we divided the data into two subsets of training and testing (about 80% and 20%, respectively). So, the frequency of ILI cases from the first week of 2010 to 25th week of 2016 was used as the training set and the rest of them were considered as the testing set. The data was scaled to the interval between [− 1, 1] before any calculations and after model building and forecasting, the data was converted to the original scale.

In the SVM, there is a need to project the input space into a feature space with higher dimension using a kernel function. Some kernel functions include Gaussian Radial Basis (GRBF), polynomial, Sigmoid, etc. [13]. In the present study we utilized the GRBF kernel $\left( {k\left( {x_{i} ,x} \right)} \right) = \exp \left( { - \gamma \left| {x_{i} - x} \right|^{2} } \right)$. When using the GRBF kernel in the SVM model, it is necessary to tune model parameters (cost that is a positive tradeoff parameter to determine the degree of the empirical error and $\gamma$) to increase the performance of the SVM. Here, we used a grid search method to find the optimum value of the parameters. So, a tenfold cross validation was conducted using the training set data partitioned into 10 subsamples randomly. Then a single subsample of the 10 subsamples is considered as the validation data for testing the model, and the remaining nine subsamples are considered as the training data. This process is then repeated 10 times and the 10 results are then averaged. Other kernels were also tried.

ANN is a flexible mathematical tool for information processing that has been widely used for forecasting and classification problems suitably that consists of input and output layers, and a hidden layer [14, 16]. A set of models based on the combination of different values for different hidden layers (from 1 to 3) were constructed to select better architecture of the MLP network. Moreover, in the hidden and output layer, the hyperbolic tangent and identity functions were used as activation functions.

#### Performance criteria

The root mean square error (RMSE), mean absolute error (MAE) and intra-class correlation coefficient (ICC) were used for evaluating the prediction accuracy of SVM, RF, ANN models. We calculated the values of sensitivity, specificity, positive predictive value (PPV), negative predictive value (NPV), and total accuracy using the following formulas [17]. All used methods were implemented using R packages [18].

### Results and discussion

The characteristics of the train and test sets were given in Table 1. According to Table 1, the statistical summaries of the train and the total data were approximately similar. For example the average weekly number of ILI cases were 24.39 (SD: 68.29) for the entire data and 25.35 (SD = 74.5) for the training data. However, the testing set was different from the training set. For the used regression methods, the RMSE, MAE and ICC statistics in training and testing sets were calculated (Table 2(a)). It is evident that the MAE (= 14.99) and RMSE (= 22.78) values for the RF time series model are smaller in testing set compared with the other two models. Moreover, the ICC (= 0.88) value related to the RF model was greater in testing set suggesting an excellent agreement between predicted and observed values of weekly ILI frequencies.

**Table 1:** The statistical parameters of monthly ILI data set

Parameter	Entire data	Training set	Test set
	2010 (first week)–2015 (52th week)	2010 (first week)–2016 (25th week)	2016 (26th week)-2018 (6th week)
Mean	24.39	25.35	20.56
Minimum	0.00	0.00	0.00
Maximum	930.00	930.00	128.00
Standard deviation	68.29	74.50	33.78
Skewness	8.05	7.69	1.88
kurtosis	87.58	76.57	2.35

**Table 2:** (a) The RMSE, MAE and ICC statistics of the used methods for prediction of ILI; (b) the performance criteria of the used methods for prediction of ILI outbreaks

(a)					
Model	Kernel		Criterion		
			RMSE	MAE	ICC
RFTS	–	Train	25.3	6.43	0.92
		Test	22.78	14.99	0.88
SVM	RBF	Train	58.71	14.3	0.58
		Test	28.19	22.36	0.53
	Polynomial	Train	55.20	15.00	0.53
		Test	239.00	91.20	0.09
	Linear	Train	53.60	13.00	0.53
		Test	30.10	18.60	0.47
	Sigmoid	Train	63.90	17.30	0.43
		Test	30.80	20.00	0.24
ANN	–	Train	37.50	11.94	0.84
		Test	26.58	13.21	0.82
ARIMA	–	Train	47.01	17.92	0.64
		Test	34.90	28.16	0.03

(b)					
Model	Sensitivity	Specificity	Criterion		
			PPV^a^	NPV^b^	Total accuracy
RF					
Train	1.000	1.000	1.000	1.000	1.000
Test	0.804	0.964	0.974	0.750	0.865
SVM					
Train	1.000	1.000	1.000	1.000	1.000
Test	0.848	0.964	0.975	0.794	0.892
ANN					
Train	0.962	0.940	0.828	0.977	0.948
Test	0.862	0.904	0.833	0.922	0.889

^a^Positive predictive value

^b^Negative predictive value

The temporal variation of the observed weekly ILI frequencies and the estimated values obtained from the three models for the test period were plotted in Fig. 1b. As can be seen, the estimated values of weekly ILI frequency were in a good agreement with their related observed values and the used models could be used to model the weekly ILI frequencies. Moreover, RF resulted in better estimated values for the observed values of ILI frequencies than the other models especially for the peak point values. Residual plots (Fig. 1c) showed that the performance of the RF model was better compared with the SVM and ANN.

The performance of the three methods in outbreaks detection (a binary variable) was also evaluated using some discriminative accuracy criteria. As shown in Table 2(b), almost all the used methods generated high specificity. Nevertheless, the sensitivity of the ANN for the test set (86.2%) was better compared to the other three methods. The total accuracy of the SVM (RBF) was 89.2% which shows excellent performance. In general, the SVM appears to be better compared with the other two methods in terms of the total accuracy. However, the performances of the three machine learning methods were almost comparable.

Early detection of the future outbreaks of ILI minimizes the impact of diseases by raising awareness of clinicians for timely diagnosis as well as treatment along with public health messaging in order to prevent high-risk behaviors/areas [12]. Performance of statistical models is data dependent and there is no model that performs well in all situations. Therefore, evaluating the performance of different methods especially those based on artificial intelligence is of great importance as they provide useful and important information regarding strengths and weaknesses of the methods [19] and gives an insight to use better models for forecasting purposes. We investigated and compared the performance of three machine learning techniques of SVM, RF and ANN in two aspects of forecasting weekly number of ILI cases with time series adaptation of them and detecting outbreaks. Our results revealed that the used machine learning techniques could be successfully used in estimating weekly ILI frequencies and outbreaks. This finding is in concordance with the results of other studies in forecasting ILI (comparing RF and ARIMA) [8, 12, 20]. Other studies evaluating the performance of machine learning time series methods in forecasting other diseases like brucellosis (comparing neural network and ARIMA) [21], gonorrhea, hemorrhagic fever renal syndrome, hepatitis A, hepatitis B, scarlet fever, schistosomiasis, syphilis and typhoid fever (comparing SVM and ARIMA) [11, 22] were also in agreement with our results confirming that the SVM and NN outperformed the ARIMA.

Our results are very worthwhile for the public health surveillance systems management and designing an automatic alarm system. Consistency and agreement between the observed and predicted data indicated a high capability of these models in modeling and estimating ILI outbreaks. In addition, these models are capable of displaying the periodic/non-periodic ILI data behavior over time. See Additional file 1 for advantages and disadvantages of the used models. As there are other hybrid methods that can improve the prediction accuracy, it is suggested to investigate other machine learning techniques in other diseases prediction as well as ILI in the future. Here we trained the model by 80% of the data and the other 20% was considered as test set (out-of-bag sample). So, we provided a relatively long-term prediction that can be different from short-term prediction and affects prediction accuracy. It is suggest that future studies investigate the accuracy of the predictions using different window sizes.

## Limitations

Weather conditions and climatic parameters including humidity, wind speed and temperature may somewhat be related to ILI. So the influence of these parameters could be used as predictors to achieve better performance of the used models. However, the used data were related to the whole country. On the other hand, Iran has a very diverse climate geographically and the weekly ILI data separated by climatic areas were not available. So, we unable to investigate the impact of these parameters. Another potential limitation of this study is sentinel based data of ILI which may affect the generalizability of the study. However, it seems sentinel data at large and national level does not affect the performance of outbreak detection tools. Reliable information about the vaccination is another important factor that may improve the performance of the used models and was not available to consider here.

## Additional file

**Additional file 1.** Advantages and disadvantages of the used models.

## Data Availability

The data is publically available on: http://www.who.int/influenza/gisrs_laboratory/flunet/en (http://apps.who.int/flumart/Default?ReportNo=12). The data is also provided as Additional file.

## Abbreviations

ILI: influenza like illness
ARIMA: autoregressive integrated moving average
SVM: support vector machine
ANN: artificial neural network
RF: random forest
KNN: K-nearest neighborhood
RMSE: root mean square error
MAE: mean absolute error
ICC: intra-class correlation coefficient
