# Supplementary material for: Comparative evaluation of time series models for predicting influenza outbreaks: application of influenza-like illness data from sentinel sites of healthcare centers in Iran
Source: BMC Res Notes. 2019 Jun 24;12:353. doi: 10.1186/s13104-019-4393-y (PMC6591835; doi:10.1186/s13104-019-4393-y)
Supplement: Supplementary file 1 — Additional file 1. Advantages and disadvantages of the used models. [file 13104_2019_4393_MOESM1_ESM.docx]

Additional file

Advantages and disadvantages of the used methods

There are different statistical approaches to detect and predict aberrations or outbreaks in a disease like ILI. Traditionally, the autoregressive integrated moving average model (a well-known time series model) has been widely used to predict future outbreaks for phenomena like diseases that their occurrence happens in a cyclic or repeating patterns (1). By filtering out high-frequency noises in the data, this model attempts to detect local trends using linear dependence in observations in the series (1). In spite of the advantages of easy interpretation and automatic model selection to maximize prediction accuracy for the ARIMA model, there are some drawbacks for this model. For example, the ARIMA considers linear relationships between independent and dependent variables which is often an unrealistic assumption and does not work well for non-linear structures. Moreover, in the ARIMA model the standard deviation of errors in the model is considered constant over time (1) and it is inefficient in long-term prediction. ARIMA does not use automatic updating feature when new data are available and there is a need to repeat the entire modeling procedure particularly the diagnostic checking stage. As there is a need to solve nonlinear estimation techniques in the ARIMA model, it tends to be unstable with respect to changes in observations and model specification.

To address the challenges associated with the classic models, machine learning techniques have been developed recently including support vector machine (SVM), artificial neural network (ANN) and random forest (RF). There are some advantages and disadvantages for the used methods in the present study. The SVM can model the nonlinearity by minimizing structural risk instead of empirical risk which will help to find the global minimum (which is unique due to the convex optimality problem) and to avoid the overfitting problem (2). However, the ANN may not be robust over different samples because of having multiple solutions associated with local minimum. The used methods have several advantages. They require less formal statistical training and can detect complex nonlinear relationships between inputs and output implicitly. They also can capture all possible interactions between inputs. Some common disadvantages in non-parametric techniques like SVM, ANN and RF are their “black box” nature that leads to the lack of transparency of results and difficult interpretations, greater computational burden and their proneness to overfitting problem. Moreover, their dimension may be very high (3, 4). For example, increasing the number of hidden layers increases the dimension of parameters dramatically. RF as one of the most accurate learning techniques can run over large databases efficiently and handle a large number of input variables without deletion. RF produces an unbiased estimate of the internal generalization error. However, like the SVM and ANN, it prunes to overfitting for noisy datasets (5, 6).

References

1. Kane MJ, Price N, Scotch M, Rabinowitz P. Comparison of ARIMA and Random Forest time series models for prediction of avian influenza H5N1 outbreaks. BMC bioinformatics. 2014;15(1):276.

2. Yoon H, Jun S-C, Hyun Y, Bae G-O, Lee K-K. A comparative study of artificial neural networks and support vector machines for predicting groundwater levels in a coastal aquifer. Journal of Hydrology. 2011;396(1):128-38.

3. Tu JV. Advantages and disadvantages of using artificial neural networks versus logistic regression for predicting medical outcomes. Journal of clinical epidemiology. 1996;49(11):1225-31.

4. Auria L, Moro RA. Support vector machines (SVM) as a technique for solvency analysis. 2008.

5. Hengl T, Nussbaum M, Wright MN, Heuvelink GB, Gräler B. Random forest as a generic framework for predictive modeling of spatial and spatio-temporal variables. PeerJ. 2018;6:e5518.

6. Tapak L, Shirmohammadi Khorram N, Hamidi O, Maryanaji ZJIJoE. Predicting the Frequency of Human Brucellosis using Climatic Indices by three Data Mining Techniques of Radial Basis Function, Multilayer Perceptron and Nearest Neighbor: A comparative study. 2018;1111:0-.
